# YOLO-UTD: A Domain-Specific Detection Framework for Small Objects in UAV Traffic Surveillance

**DOI:** 10.3390/s26123931

**Published:** 2026-06-20

**Authors:** Hailang Huang, Meng Li, Jiebao Zhang, Yitong Li

**Affiliations:** 1 School of Civil Engineering, Tsinghua University, Beijing 100084, China; hl-huang22@mails.tsinghua.edu.cn; 2 JSTI Group, Nanjing 210019, China; zjb@jsti.com (J.Z.); lyt606@jsti.com (Y.L.)

**Keywords:** traffic surveillance, UAV vision, small-object detection, YOLOv8, feature pyramid network

## Abstract

Detecting objects in drone-captured aerial imagery is particularly formidable due to challenges such as the prevalence of numerous small targets and their dense spatial distribution. To bridge this gap, this paper introduces YOLO-UTD (YOLO-UAV Traffic Detection), a dedicated small object detector tailored for drone traffic surveillance. Built upon the YOLOv8 framework, the proposed model incorporates three principal enhancements. First, a specialized small-object detection head replaces the original large-object head to increase the sensitivity to fine-grained features. Second, we introduce a shallow-augmented feature pyramid network (SFPN) into the neck module. The SFPN enriches the semantic content of high-resolution shallow features via dense multiscale interactions and CARAFE upsampling, boosting performance on small targets. Finally, a C2fA layer is integrated into the deep backbone stages to adaptively fuse spatial details and semantic context through a dual-path architecture and a cross-attention mechanism, thereby dynamically refining features critical for small objects. Extensive experiments on the VisDrone2019 dataset validate that YOLO-UTD achieves a 3.6% higher mean average precision (mAP) than YOLOv8 while preserving a low parameter footprint, with a particularly significant gain of 5.3% in vehicle detection accuracy. These findings confirm the model’s efficacy and strong potential for application in smart city drone surveillance.

## 1. Introduction

The accelerating pace of urbanization and rising transportation demands impose severe challenges on traffic system efficiency and safety. Frequent congestion and accidents compromise travel experience and public safety. Unmanned aerial vehicles (UAVs, or drones) present a promising technological solution for traffic monitoring, capitalizing on their high mobility, broad field of view, and flexible deployment [1]. Equipped with high-resolution cameras, drones efficiently capture large-scale traffic imagery, enabling applications in real-time monitoring, accident forensics, and violation documentation.

Nevertheless, object detection in drone-captured traffic scenes remains challenging due to the unique overhead perspective and complex scene characteristics. Compared with conventional ground-based traffic monitoring, UAV imagery presents two prominent domain-specific hurdles that further exacerbate detection difficulty. First, objects are extremely small and dominant: more than 80% of targets occupy no more than 32 × 32 pixels, with limited discernible features, a scale rarely encountered in ground views. Second, targets are densely distributed and frequently occluded, while backgrounds are highly cluttered with urban textures, buildings, and vegetation that severely interfere with feature extraction—unlike the relatively structured and clean fields of view of fixed ground cameras. Together, these unique traits severely degrade the performance of generic detectors, which lack specialized optimization for the extreme small-target ratio and complex background interference in aerial traffic scenes.

To advance drone-based traffic detection, especially for small objects, substantial research efforts have been devoted. The mainstream detectors fall into two categories: single-stage models such as the YOLO series and two-stage models such as the R-CNN series. We adopt YOLOv8 as our baseline because of its favorable accuracy–speed trade-off, robust codebase, and prevalent industrial adoption. However, YOLOv8 struggles with the extremely small size and high density of objects in drone imagery. The performance of existing YOLOv8 refinements remains inadequate for small object detection, notably in the complex, traffic-rich environments encountered by drones.

To address this gap, a novel framework termed YOLO-UTD is proposed. Systematic enhancements to the detection head, neck, and backbone of YOLOv8 are introduced, specifically tailored for UAV traffic scenarios. Experiments confirm that YOLO-UTD achieves superior accuracy over the baseline, establishing a solid foundation for real-time aerial traffic inspection.

Specifically, this work targets UAV-based traffic monitoring at altitudes of 60–120 m over urban arterial roads and intersections. The primary objects of interest are vehicles (cars, vans, trucks, buses) and pedestrians, as they are most critical for traffic management. Bicycles and other small categories are also evaluated but are inherently more challenging due to their very small pixel size and frequent occlusion.

**Figure 1:**
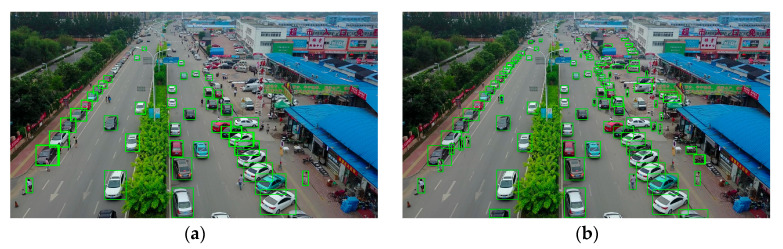
Comparison of inference results between YOLOv8n and YOLO-UTD. (**a**) Detection results of YOLO-UTD; (**b**) Detection results of YOLOv8n.

This paper makes the following core contributions:

- A specialized small-object detection head. We replace the original large-object detection head in YOLOv8 with a dedicated design for small objects. This modification directly enhances the model’s capacity to capture fine-grained features and textures, yielding a substantial gain in sensitivity to small drone targets.
- Shallow-augmented feature pyramid network. To better leverage the underutilized high-resolution shallow features, we design an SFPN that places shallow features at its core. The network employs dense multiscale feature interaction alongside a CARAFE upsampling module [2], which collectively preserves and enriches fine details within the feature pyramid, leading to significant gains in detecting small UAV targets.
- A backbone enhancement module (C2fA) for tiny objects. We introduce a C2fA layer into the deep stages of the backbone, which integrates the EMA mechanism [3] into the standard C2f block. Through a dual-path structure and a cross-attention mechanism, this layer adaptively fuses spatial details with the semantic context, thereby reinforcing features pertinent to tiny objects and mitigating the issue of feature degradation or loss for small objects in deep networks.
- Comprehensive model integration and validation. The synergistic integration of the above components constitutes YOLO-UTD, a model tailored for drone traffic scenes. An extensive evaluation of the VisDrone2019 dataset [4] revealed that YOLO-UTD outperforms the baseline by 3.6% in terms of mAP and 5.2% in terms of vehicle detection accuracy while maintaining inference efficiency. As illustrated in Figure 1, YOLO-UTD (a) identifies significantly more small objects in cluttered backgrounds than YOLOv8n (b), demonstrating its superior effectiveness and providing a robust technical solution for smart city drone surveillance.

## 2. Related Work

Owing to their high mobility and low operational cost, UAVs have become invaluable for intelligent traffic management and large-scale surveillance, offering novel solutions for monitoring tasks. However, object detection in drone-captured aerial imagery, particularly for small objects, remains a formidable challenge. The primary difficulties stem from the unique aerial perspective: significant scale variation due to changing altitude and viewpoint; a minuscule pixel footprint and consequently weak feature representation for distant objects (e.g., vehicles, pedestrians), leading to feature degradation in deep networks; a lack of distinctive textures and limited discriminative information, making small objects prone to being subsumed by cluttered urban backgrounds; and frequent occlusion or crowding. These factors collectively hinder accurate detection [5,6,7,8,9].

Small objects pose a long-standing and formidable challenge in the field of computer vision. The initial phase of development was dominated by the two-stage paradigm, with Fast R-CNN [10] and Faster R-CNN being primary examples. The multistep pipeline of these frameworks, particularly the RPN and ROI pooling, leads to substantial feature map information loss—a major bottleneck for small object detection. This occurs despite their commendable accuracy, achieved through the canonical sequence of region proposal generation, classification, and bounding-box regression. Moreover, their computational complexity and slow inference speed make them unsuitable for real-time applications such as drone surveillance. Operating in a single forward pass, single-stage detectors perform direct prediction of bounding boxes and class probabilities from the input image. This streamlined pipeline enables efficient, end-to-end learning and inference. This paradigm shift offers a critical advantage in speed and has effectively mitigated many limitations of two-stage methods for real-time scenarios.

Among single-stage object detectors, the YOLO family stands out as a prominent and continuously evolving representative. The seminal YOLOv1 [11] pioneered a regression-based framework for end-to-end detection. Its successors introduced key innovations: YOLOv2 [12] incorporates batch normalization and anchor boxes; YOLOv3 [13] leverages multiscale feature fusion; and YOLOv4 [14] integrates advanced data augmentation, model optimization, and head enhancements. Subsequent versions, including YOLOv5, v6 [15], and v7 [16], further refine the architecture and training pipeline for improved efficiency and performance. Notably, YOLOv8 introduces an optimized feature fusion structure and other enhancements, which bolster its ability to detect small objects.

Driven by the need for aerial scene analysis, recent research has tailored YOLO architectures specifically for small objects. For example, ESOD-YOLOv8 [17] integrates automatic disturbance resistance convolution (ADRConv) to better extract small-object features and suppress background interference. Lou et al. [18] extended ESOD-YOLOv8 by establishing lateral and longitudinal connections between feature maps, thereby enriching their multiscale representations. Temporal-YOLOv8 [19] improves accuracy for small object detection by incorporating multiframe temporal cues. LSOD-YOLO [20] pursues a lightweight design for edge deployment. Other notable variants, such as YOLOv8s-SOD [21] and the more recent YOLO-10/11/12 series [22,23], continue to increase the accuracy and efficiency of boundaries. Autonomous driving, security surveillance, and medical imaging are among the domains where YOLO-based models are widely applied, due to their favorable speed–accuracy trade-off. To address the conflict between the progressive reduction in feature map resolution and the preservation of small object details in deep convolutional neural networks, multiscale feature fusion has emerged as a key technique. The feature pyramid network (FPN) proposed by Lin et al. [24] combines high-level semantic information with high-resolution shallow features through a top-down pathway, effectively enhancing the network’s representational capacity for small objects. The path aggregation network (PANet) [25] subsequently augmented the FPN by adding a bottom-up pathway, further facilitating the transmission of shallow positional information to higher layers. BiFPN [26] simplified the architecture and achieved efficient bidirectional fusion of multiscale features by introducing learnable weights, becoming a standard component in the necks of many modern detectors. Despite the effectiveness of these multiscale fusion strategies, semantic gaps and noise issues may persist during the fusion process.

To counteract the weak and fragmented features characteristic of small objects, attention mechanisms guide model focus to salient regions. Channel-wise refinement, as seen in the squeeze-and-excitation (SE) module [27], addresses this by adaptively recalibrating feature responses across channels. Further integration of spatial cues is achieved through dual-attention mechanisms like CBAM [28], which sequentially applies both channel and spatial attention to refine feature maps.

Beyond these feature-level recalibrations, incorporating broader contextual information forms another essential strategy for small object detection. The atrous spatial pyramid pooling (ASPP) module [29] captures multiscale context through parallel atrous convolutions, enhancing feature representation. Global context networks, such as GCNet [30], model long-range dependencies between objects and the background to improve performance. Furthermore, transformer-based approaches have been explored; for example, Wang et al. [31] employed a global attention mechanism to model intricate semantic relationships in scene graphs, thereby increasing detection accuracy.

## 3. Methodology

**Figure 2:**
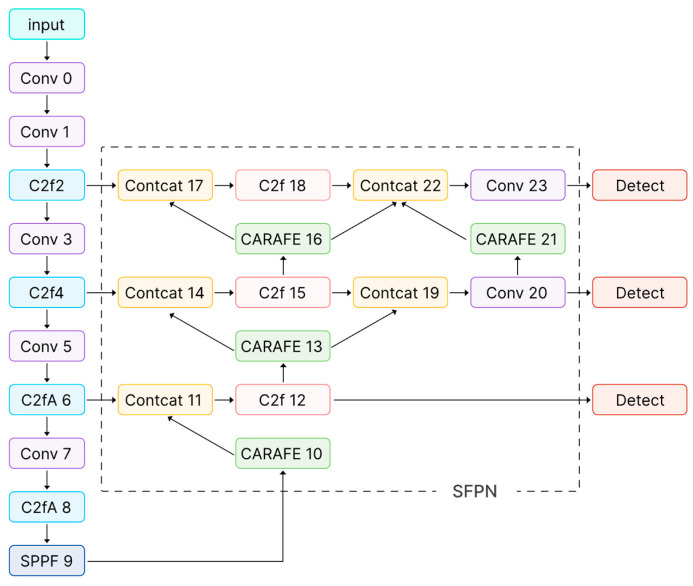
YOLO-UTD Model’s Structural Diagram.

This section presents YOLO-UTD, a model designed to address the suboptimal detection performance for small, low-resolution objects typical of UAV imagery. Our framework builds upon YOLOv8 and introduces targeted enhancements across three core components: the backbone, neck, and detection head. These synergistic modifications collectively increase the model’s sensitivity and discriminative power for small targets. The overall architecture is depicted in Figure 2. The rationale and technical details of each enhanced module are described in subsequent subsections.

### 3.1. Model Head Improvement

In aerial traffic scenarios dominated by wide-angle lenses and long imaging distances, aiming at minute object detection, we first introduce a dedicated tiny-object detection head with a 160 × 160 resolution. This head connects to shallow backbone layers (stride 4), which preserves richer spatial details and high-resolution texture features, thereby mitigating the feature degradation that typically occurs in deep networks for small objects. Through high-density anchor sampling, the design sharply increases the model’s perceptual acuity for near-pixel-level targets, establishing a structural basis for high recall and precise localization.

Furthermore, we reconsider the role of the original YOLOv8 20 × 20 large-object detection head, which is redundant for UAV traffic scenes. In our typical operating altitude of 60–120 m, large objects are effectively absent, and even the largest targets fall below the effective detection threshold of this head. Retaining it would only introduce unnecessary parameters, extra computation, and hard negative samples that hinder training. We therefore remove the 20 × 20 large-object head entirely and introduce a dedicated 160 × 160 P2 tiny-object detection head tailored for UAV imagery, where targets are extremely small in pixel size. This high-resolution head preserves rich shallow spatial details critical for tiny aerial objects—a design unnecessary for ground surveillance systems that mainly focus on large or medium targets. By this design, we reallocate model capacity exclusively to small- and medium-object feature learning, eliminating latency and noise while enhancing sensitivity to minute targets.

### 3.2. Shallow Enhanced Feature Pyramid Network

The original YOLOv8 architecture is limited in handling small objects from drone views because of progressive information loss in its feature extraction process. As features propagate deeper, their spatial resolution is reduced, discarding fine-grained details crucial for small targets. Although deeper layers benefit from a larger receptive field, they often fail to preserve the precise textures and contours of minute objects. This issue is particularly severe in UAV imagery, where shallow high-resolution features carry far more critical information for tiny aerial targets than in general scenes. To counteract this, we redesign the neck network and propose the SFPN.

**Figure 3:**
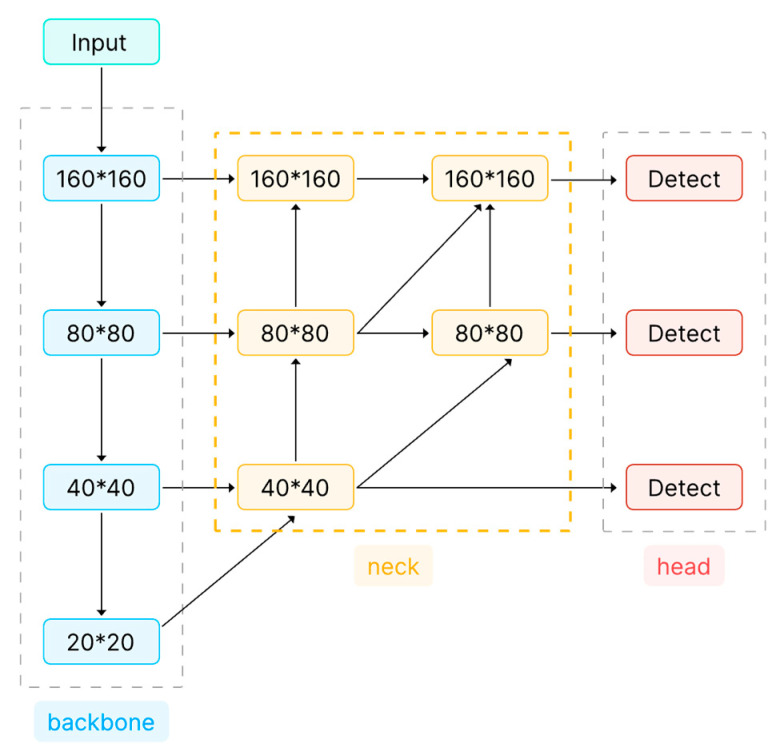
Schematic of the YOLO-UTD neck structure. Numbers within each block denote the resolution of the corresponding feature maps.

The standard YOLOv8 neck uses a PANet for multiscale feature aggregation via bidirectional connections; however, it primarily enriches deep semantic information at the expense of underutilizing high-resolution shallow features. Given the predominance of small targets in our setting, the fine details in shallow features are vital. Our SFPN, therefore, builds upon an FPN backbone to prioritize and reinforce shallow feature representation and fusion. Specifically, we retain the P4 (40 × 40) feature map for medium-sized object detection. This map is also upsampled and concatenated with the P3 (80 × 80) map; after convolution, the resulting features are directed to the small-object detection head. To further empower tiny object detection, we densely integrate multiscale information by concatenating the P3 (80 × 80) feature from the FPN, a newly introduced high-resolution P2 (160 × 160) feature map, and the processed 80 × 80 features from the small-object detection path. A final convolution on this concatenated output feeds the tiny-object detection head. This design fosters dense cross-scale feature interactions, substantially boosting the perception and localization of tiny objects. The overall SFPN architecture is illustrated in Figure 3.

The neck of the original YOLOv8 employs nearest neighbor interpolation for its upsampling operations. This method assigns weights based solely on fixed pixel distances, resulting in a limited, content-agnostic receptive field. Consequently, substantial detail loss occurs during feature fusion, hampering precise localization especially for small targets. In aerial scenes, such detail blurring is far more harmful to tiny object detection; we therefore integrate the lightweight content-aware upsampler CARAFE to replace the original interpolation scheme, effectively preserving fine details for UAV small targets.

CARAFE overcomes these limitations through the synergistic operation of its two modules. The kernel prediction module (defined in Equation (1)) works in tandem with the content-aware reassembly module to achieve upsampling that is both content-aware and possesses a large receptive field.

(1) $$W_{upsampling}=softmax(shuffle(conv_{encoder}(conv_{1\times 1}(I))))$$

The kernel prediction module first applies a 1 × 1 convolution to the input feature map I (shape: *H* × *W* × *C*) for channel compression and content encoding, outputting a compressed feature map of size *H* × *W* × *C_m_*. This compressed map is further processed to predict the upsampling kernels, generating an encoded feature of size *H* × *W* × (*σ*^2^ × *C*_encoder_). A shuffling operation transforms this tensor into a tensor of size *σH* × *σW* × *C*_upkernel_^2^, where *C*_upkernel_^2^ denotes the channel count corresponding to the upsampling kernel of shape *upkernel* × *upkernel*, and *σH* × *σW* specifies the spatial dimensions of the upsampled feature map. The kernels are then normalized across channels using a softmax function, yielding the final upsampling prediction kernels *W*_*upsampling*_.

**Figure 4:**
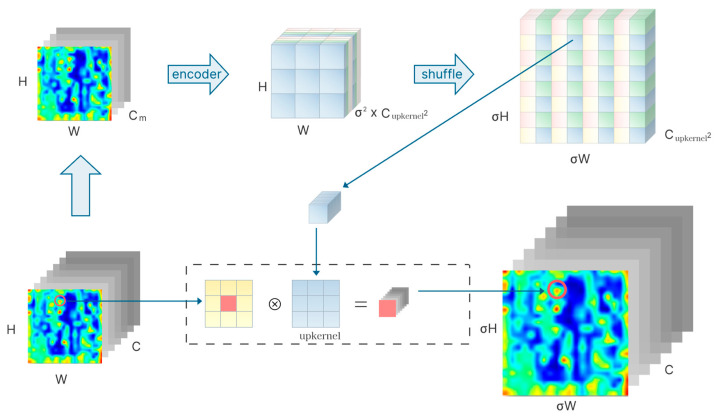
Specific operational workflow of the CARAFE upsampling module.

The process begins with appropriately zero-padding the input feature map. Subsequently, the content-aware reassembly module proceeds with upsampling. For each target location in the upsampled output, a sliding window of size *upkernel* × *upkernel* extracts the corresponding local region from the feature map. A dot product is computed between this region and its content-adaptively predicted kernel. The resulting values are reshaped to form the upsampled feature map. Finally, a 1 × 1 convolution adjusts the channel dimension back to the original count, completing the operation. The workflow of CARAFE is illustrated in Figure 4.

In synergy, the kernel prediction module dynamically generates a content-specific upsampling kernel for each location, guaranteeing that the upsampling process is fully adaptive to local image structures. The feature reassembly module then leverages these kernels to perform a weighted recombination of features over a large receptive field. This content-aware mechanism allows CARAFE to effectively utilize contextual cues for high-fidelity feature reconstruction, restoring resolution with richer detail and superior semantic consistency.

Integrating CARAFE provides two principal advantages. First, it substantially boosts the model’s capacity to reconstruct features for tiny objects—a critical capability for detecting vehicles and pedestrians that occupy minimal pixel areas in drone imagery. Second, CARAFE remains lightweight, adding negligible parameters and computational overhead. This ensures that the model satisfies the stringent efficiency constraints of drone platforms without compromising performance gains. Collectively, this enhancement allows the neck network to preserve and refine the fine details of small objects during multiscale fusion, creating a powerful synergy with the newly introduced tiny-object detection head.

### 3.3. Adding Attention Modules to the Backbone

The YOLOv8 backbone extracts multilevel, robust feature representations from input images. Its core C2f module effectively aggregates features across receptive fields via dense cross-layer connections; however, it often fails to adequately represent the subtle visual cues of the extremely small targets prevalent in drone imagery. To bolster the network’s capacity to capture and retain tiny object features, we augment the C2f module by embedding an efficient multiscale attention (EMA) mechanism, forming our proposed C2fA block. The EMA employs a lightweight, groupwise design to construct parallel spatial and channel attention pathways, sharply boosting sensitivity to critical information with minimal computational cost.

**Figure 5:**
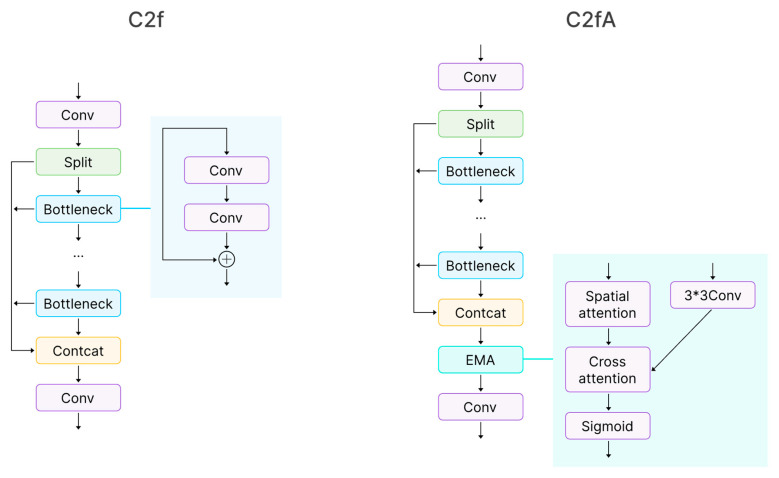
The C2f structure is shown on the left and the C2fA module structure diagram on the right.

As illustrated in Figure 5, the EMA mechanism operates through a structured pipeline. The feature map first undergoes partitioning into *G* channel groups, followed by spatial compression across its height and width to yield orientation-aware features. These features are then fused across orientations via a 1 × 1 convolution to generate spatial attention weights. The features are subsequently processed through two parallel pathways: one branch integrates the spatial weights with the original features, followed by group normalization, whereas the other branch extracts local structural patterns via a 3 × 3 convolution. These pathways engage in mutual calibration through a cross-attention mechanism, where each branch generates channel attention weights via global average pooling, which are applied to weight the features of the complementary branch. Finally, the refined features from both pathways are aggregated through sigmoid-weighted fusion to produce the final enhanced feature map. The computation of spatial attention weights is formalized in Equation (2):

(2) $$\left\{\begin{array}{l} X_{group}=reshape(X,[B\times G,C//G,H,W])\\ X_{h}=Avg_{\left(H,1\right)}(X_{group})\\ X_{w}=Avg_{\left(1,W\right)}(X_{group})\\ H_{c}=Conv_{1\times 1}(Concat(X_{h},X_{w}))\\ W_{h},W_{w}=Split(H_{c},[H,W])\\ X_{1}=GN(X_{group}⊗\sigma (W_{h})⊗\sigma (W_{w}))\\ X_{2}=Conv_{3\times 3}(X_{group}) \end{array}\right.$$

In the above formulations, *B*, *G*, *C*, *H* and *W* denote the batch size, grouping number, total input channels, spatial height and width of input feature *X*, respectively. “*reshape*” denotes a grouping operation; *Avg*_(*H*,1)_ and *Avg*_(1,*W*)_ represent average pooling applied along the height and width dimensions, respectively; *Conv*_1×1_ and *Conv*_3×3_ correspond to 1 × 1 and 3 × 3 convolutions; Concat indicates feature concatenation; Split refers to splitting along the channel dimension; ⨂ signifies elementwise multiplication; and *GN* is Group normalization. Equation (2) implements dual-path spatial feature modulation to extract height-wise and width-wise attention cues, yielding two intermediate features *X*_1_ and *X*_2_.

The operations governing the cross-attention mechanism and the subsequent weighted fusion are formally defined in Equation (3):

(3) $$\left\{\begin{array}{l} A_{1,2}=Softmax(GAP(X_{1,2}))\\ F_{1,2}=reshape(X_{1,2})\\ W=sigmoid(A_{1}\cdot F_{1}+A_{2}\cdot F_{2})\\ Y=X_{group}⊗W \end{array}\right.$$

In this context, *Softmax* refers to the Softmax normalization function, *GAP* denotes global average pooling, and sigmoid is the sigmoid activation function. The first two equations derive channel attention weights *A*_1_ and *A*_2_ from *X*_1_ and *X*_2_, while the third equation fuses them via weighted summation and sigmoid to form a final attention map *W*. The output *Y* is then computed as the element-wise product of *X_group_* and *W*, achieving adaptive feature recalibration.

We construct the new C2fA module by integrating the EMA module into the backbone’s C2f block. Its detailed architecture is presented on the right side of Figure 5. Crucially, we replace only the C2f modules at the deepest stages—specifically those with strides of 16 and 32—with C2fA. This selective placement is motivated by the distinct characteristics of deep versus shallow features. Deep features, while rich in semantics and channel capacity, suffer from severe degradation of spatial details for small objects after repeated downsampling. The EMA module’s dual-path design is strategically deployed here to counteract this loss: its spatial attention path recalibrates the attenuated feature responses of small targets, whereas its cross-attention path fosters dynamic interplay between channelwise semantics and spatial context. This hierarchical enhancement strategy establishes a tiered refinement system: it circumvents the potential noise amplification that could arise from applying attention in shallow layers while guaranteeing that deep features are maximally refined before propagation to subsequent detection stages. Consequently, the backbone supplies more discriminative feature maps to the neck and detection heads, directly improving small-object detection.

## 4. Experiments

### 4.1. Dataset

The VisDrone2019 dataset was utilized in our experiments. This standard evaluation resource for drone-captured imagery was originally released by the AISKYEYE development team. Captured across 14 Chinese cities under diverse weather and lighting by various UAV platforms, the dataset comprises 10,209 real-world images (training: 6471; validation: 548; testing: 1610). With over 2.6 million high-quality bounding box annotations across 10 categories, it provides a rigorous testbed for evaluating robustness in complex scenarios. Critically, the dominant top-down perspective results in most targets occupying minuscule pixel areas, making VisDrone2019 an authoritative benchmark for small object detection.

### 4.2. Experimental Environment

All experiments were performed on an Ubuntu 24.04.1 system utilizing Python 3.8.10, PyTorch 2.1.0, and CUDA 11.8. The hardware platform included an NVIDIA GeForce RTX 2080 Ti GPU. We built our models on the official Ultralytics YOLOv8 framework, implementing the proposed architectural modifications. For fair and reproducible comparisons, consistent hyperparameters were used across all training and evaluation phases. The models were trained for 300 epochs with the input images resized to 640 × 640 and initialized from the official YOLOv8n.pt pretrained weights. The inference speed was measured via a single image. All experiments in this paper, including ablation studies and comparative evaluations, are conducted on the official VisDrone2019 test set (1610 images).

### 4.3. Comparative Experiments

To validate the efficacy of our approach, we first benchmark YOLO-UTD against several state-of-the-art detectors, including YOLOv8n, YOLOv10n, YOLOv11n, YOLOv12, RT-DETR [32], RT-DETR-V4 [33], Mamba-YOLO [34] and DEIM [35] under identical experimental settings. As summarized in Table 1, YOLO-UTD achieves the best performance on key metrics such as mAP@50 and mAP@50:95, demonstrating clear advantages over the baseline models. The highest scores are highlighted in bold.

As listed in Table 1, all variants of the proposed YOLO-UTD consistently outperform mainstream lightweight detection models on the VisDrone2019 aerial benchmark in terms of accuracy–efficiency balance.

Taking the smallest YOLO-UTD-n as an example, it obtains 32.6% mAP@50, which surpasses the YOLOv8n baseline by an absolute margin of 3.5%. Benefiting from our optimized network design, YOLO-UTD-n reduces total parameters by approximately 12.6% compared with YOLOv8n, verifying superior parameter utilization efficiency. Despite a mild 5.1 ms increase in per-frame inference delay, the substantial accuracy gain compensates for this slight computational cost, realizing a competitive precision-speed trade-off.

In the medium-size setting, YOLO-UTD-s is compared with RT-DETR-V4-S with close parameter scale. The two models deliver nearly identical detection accuracy: 37.3% versus 37.7% for mAP@50 and 21.7% versus 21.6% for mAP@50:95. Nevertheless, YOLO-UTD-s achieves this competitive accuracy with fewer parameters (9.76 M vs. 10.23 M) and drastically lower inference latency (16.4 ms, only 49.4% of RT-DETR-V4-S’s 33.2 ms). For UAV aerial detection scenarios with rigid real-time and onboard edge-computing limitations, the prominent speed edge of YOLO-UTD greatly enhances its engineering practicability for real-world UAV deployment. Moreover, the gradually lifted mAP metrics from YOLO-UTD-n → s → m → l → x validates the good scalability of our architecture across different model scales. The scalability experiments for different model sizes will be specifically analyzed in Section 4.5.

It is also worth noting that, when compared with the similarly sized RT-DETR-V4-S, our YOLO-UTD-s achieves nearly identical accuracy (37.3% vs. 37.7% mAP@50, and 21.7% vs. 21.6% mAP@50:95) with fewer parameters (9.76 M vs. 10.23 M) and, more notably, less than half the inference latency (16.4 ms vs. 33.2 ms). Given the stringent real-time requirements of UAV systems, this significant speed advantage further.

For a fine-grained analysis, Table 2 presents the per-category mAP@50 scores of all compared methods on the ten classes of the VisDrone2019 test dataset. Models with excessive parameter counts would introduce redundant comparisons in this section; therefore, only the n- and s-scale variants of YOLO-UTD are presented here.

The results in Table 2 confirm that YOLO-UTD consistently outperforms the baseline across all object categories, underscoring its robust generalizability. A particularly notable gain is observed for the vehicle class, where the accuracy increases from 68.1% to 73.4%, an improvement of 5.3 percentage points.

In summary, YOLO-UTD delivers significantly higher detection accuracy with fewer parameters while maintaining inference latency nearly on par with the baseline. These results collectively validate the efficacy of our architectural enhancements.

### 4.4. Ablation Study

We conduct ablation studies to evaluate the impact of each key component in YOLO-UTD. Starting from the YOLOv8n baseline, we progressively incorporate our proposed modules, including the specialized detection head, SFPN neck, and C2fA backbone enhancement. Each addition is assessed on the VisDrone2019 test set to determine its contribution to the overall performance.

#### 4.4.1. Head Ablation Experiments

Our ablation study first examines the influence of the detection head configuration. Table 3 uses the notation P5, P4, P3, and P2 to denote detection heads responsible for large, medium, small, and tiny objects, respectively.

Our stepwise ablation study, detailed in Table 3, provides several key insights. Integrating a tiny-object detection head (P2, 160 × 160) into the baseline model results in a notable increase in mAP@50 from 29.1% to 29.7%, confirming its effectiveness in capturing minute features. Further refinement involves removing the large-object head (P5, 20 × 20), which leads to an additional improvement in mAP@50 to 30.8%. This indicates that eliminating this redundant component allows the model to focus more effectively on the small- and medium-sized targets that are common in aerial imagery. However, when both the large- and medium-object heads (P4, 40 × 40) are removed, the performance decreases to 30.5%, highlighting the essential role of the medium-object head in maintaining detection accuracy. In conclusion, the optimal configuration is achieved by retaining the medium-object head (P4), removing the large-object head (P5), and adding the tiny-object head (P2). This setup strikes the best balance between accuracy and model complexity.

#### 4.4.2. Neck Ablation Experiments

Our study assesses the effects of varying neck architectures on detection accuracy by employing the optimized model as the new baseline. Table 4 shows the experimental outcomes.

Starting from the optimized detection head configuration (30.8% mAP@50), we first replaced the original PANet with a bidirectional FPN (BiFPN). This change led to a decrease in the mAP@50 to 30.0%. We hypothesize that although BiFPN efficiently fuses multiscale features, its architecture does not sufficiently prioritize the high-resolution, fine-grained details from the newly added 160 × 160 tiny-object feature map, leading to suboptimal use of shallow information.

Replacing PANet with a standard FPN improved the accuracy to 31.4%, indicating that a simpler, top-down fusion path is more effective for our task. Ultimately, our proposed SFPN achieves the best performance, with a mAP@50 of 32.2%. This validates SFPN’s superior capability in multiscale feature integration, especially its effectiveness in preserving and enhancing shallow, high-resolution details critical for small object detection in aerial imagery.

#### 4.4.3. Backbone Ablation Experiments

Finally, we evaluate the effect of embedding the C2fA module at different depths within the backbone. Using the model with an optimized head and SFPN neck (32.2% mAP@50) as the baseline, we compare three placement strategies for C2fA, as detailed in Table 5:

The ablation results, as shown in Table 5, can be summarized as follows. Replacing all four C2f layers with C2fA leads to an mAP@50 of 32.4%, a slight increase from the baseline of 32.2%, whereas the mAP@50:95 remains unchanged at 18.3%. When C2fA is applied only to the two shallow C2f modules, the mAP@50 decreases slightly to 32.1%, indicating that introducing attention mechanisms at early stages does not yield benefits and may be due to interference from noisy, low-level features. In contrast, applying C2FA solely to the two deep C2f modules achieves the best performance, with the mAP@50 increasing to 32.6% and the mAP@50:95 improving to 18.5%. These results confirm our design philosophy: implementing C2fA in deeper layers allows optimal use of its cross-attention mechanism to refine high-level semantic features, which is critical for distinguishing small objects. Consequently, our final architecture adopts the strategy of replacing only the deep C2f modules with C2fA.

#### 4.4.4. YOLO-UTD Ablation Study

To evaluate the impact of every proposed component, we carried out a comprehensive ablation study on YOLO-UTD. The component-wise performance results are presented in Table 6.

Table 6 summarizes the progressive improvements achieved by incrementally integrating our proposed modules into the YOLOv8n baseline (3.01 M parameters, 0.291 mAP@50, 0.167 mAP@50:95).

First, optimizing the detection head—adding a tiny-object head (P2) and removing the large-object head (P5)—boosts mAP@50 to 0.308 (+1.7%) and mAP@50:95 to 0.176 (+0.9%). By reducing the number of parameters substantially to 2.01 M, our method demonstrates that head specialization not only streamlines model complexity but also leads to enhanced detection performance for small objects.

Subsequently, integrating SFPN and CARAFE further increases the performance to 0.322 mAP@50 (+1.4%) and 0.183 mAP@50:95 (+0.7%), with a moderate parameter increase to 2.59 M. The gain validates the SFPN’s efficacy in enhancing multiscale detection through improved shallow-feature propagation and fusion.

Finally, incorporating the C2fA module into the deep backbone stages yields the complete YOLO-UTD, achieving the best results: 0.326 mAP@50 (+0.4%) and 0.185 mAP@50:95 (+0.2%), with 2.63 M parameters. This step refines high-level feature representation, providing the final performance margin.

It is worth noting that the improvement brought by C2fA, though modest in absolute terms (+0.4% mAP@50), comes at an extremely low cost: only 0.03 M additional parameters and 2.7 ms extra inference time, keeping the overall latency well within real-time requirements for UAV deployment (15.9 ms vs. 30 ms threshold). We also explored simpler attention alternatives in place of EMA within the deep C2f layers; these either produced no measurable gain or even degraded accuracy. Hence, the marginal yet stable improvement from C2fA is non-redundant and justifies its inclusion, especially given the inherent difficulty of detecting small objects in aerial imagery.

In summary, the ablation study validates the distinct and complementary roles of each component: head optimization provides an efficient performance baseline, SFPN strengthens multiscale feature integration, and C2fA enhances semantic feature quality. The synergistic combination of these methods drives the overall advancement of YOLO-UTD.

### 4.5. Scalability Experiments Across Different Model Sizes

To demonstrate the generality and scalability of our improvements across model scales, we conduct consistent experiments on five variants of the YOLOv8 series: YOLOv8n, YOLOv8s, YOLOv8m, YOLOv8l, and YOLOv8x (spanning from nano- to extra-large). For each variant, we compare the original model against its improved counterpart (denoted as “improvement”), with the results detailed in Table 7.

The results in Table 7 support two key observations:

Consistent Performance Gains Across Scales: Our improvements deliver consistent accuracy gains for all model sizes. In terms of mAP@50, the enhanced models exhibit absolute improvements of 3.2% to 4.4% over their respective baselines. This confirms that our modules constitute a generalizable enhancement strategy rather than an overfit to a specific model capacity, successfully transferring across architectures of varying complexity.

Greater Relative Gains for Lightweight Models: The relative performance improvement is most pronounced for lightweight models. For example, the nano (n) and small (s) variants achieve relative mAP@50 gains of 12.41% and 13.37%, respectively, whereas the large (l) and extra-large (x) variants yield gains of 7.07% and 8.31%, respectively. We attribute this to the inherent limitations in feature representation of compact models; their constrained capacity makes them more receptive to our targeted optimizations, thus yielding higher marginal returns.

### 4.6. Supplementary Experiments on UAVDT Dataset

To further evaluate the generalizability of YOLO-UTD beyond VisDrone2019, we conducted additional experiments on the UAVDT [36] (Unmanned Aerial Vehicle Benchmark for Object Detection and Tracking) dataset. UAVDT contains aerial images captured under various urban scenarios, with rich annotations for vehicles (car, truck, bus). We compare YOLO-UTD against the baseline YOLOv8n under identical experimental settings. The results are summarized in Table 8.

As shown in Table 8, YOLO-UTD achieves a significant improvement of +7.6% in mAP@50 over the baseline, while reducing the parameter count by approximately 12.6%. The consistent gains on both VisDrone2019 and UAVDT datasets demonstrate that our proposed modules are not overfitted to a single benchmark but provide robust and generalizable enhancement for small object detection in UAV traffic surveillance.

### 4.7. Visualization Results Analysis

**Figure 6:**
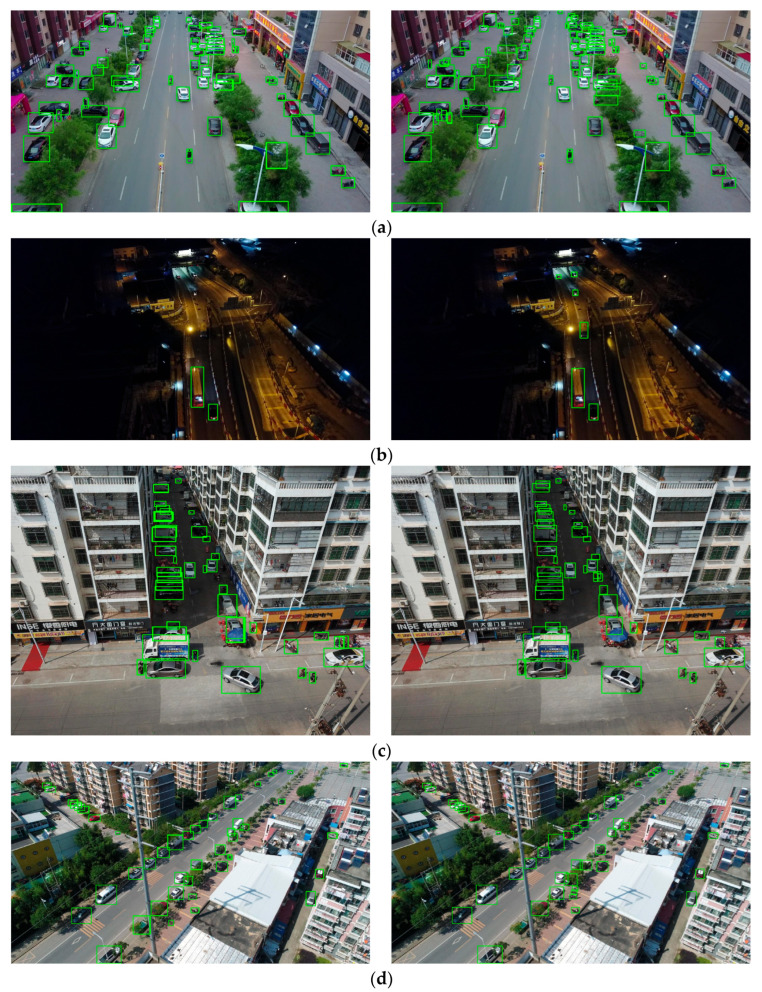
Comparison of inference results between the baseline algorithm and YOLO-UTD. The left side shows the inference results of the original YOLOv8 source code, whereas the right side displays the inference results of YOLO-UTD. (**a**): long-range scenes; (**b**): low-light conditions; (**c**,**d**): cluttered scenes.

Figure 6 provides a visual comparison of the detection results between YOLO-UTD and the baseline YOLOv8n across representative challenging scenarios, offering qualitative validation of our method’s performance. The visual comparisons reveal marked advantages for YOLO-UTD.

Specifically, in long-range scenes (Figure 6a), YOLO-UTD detects more distant small objects (e.g., vehicles, pedestrians) missed by the baseline, underscoring its improved sensitivity to tiny features. Under low-light conditions (Figure 6b), it maintains robust detection of distant, low-contrast objects, successfully identifying targets in dark areas overlooked by YOLOv8n. Finally, in cluttered scenes (Figure 6c,d), YOLO-UTD yields multifaceted gains: it produces bounding boxes with higher localization precision (reducing overlap and redundancy) and effectively suppresses false positives in complex backgrounds while detecting small objects.

## 5. Conclusions

This work addresses the critical problem of small-object detection in drone-captured aerial imagery and presents YOLO-UTD—an enhanced and efficient model built upon YOLOv8. We introduce three principal improvements: (1) optimizing the detection head by replacing the large-object head with a dedicated tiny-object head to better capture minute features; (2) designing the SFPN integrated with the CARAFE upsampler, which markedly improves the preservation and fusion of fine-grained details; and (3) incorporating C2fA modules into the deep backbone stages to strengthen the joint representation of semantic context and spatial information.

Future work will pursue three directions: First, further optimizing computational efficiency via lightweight architectures for edge deployment. Second, improving cross-scene generalization to maintain robust performance under diverse environmental conditions (e.g., varying illumination and weather). Third, enhancing detection for extremely small categories such as bicycles, people, tricycles, and awning tricycles by incorporating temporal context from video sequences and higher-resolution input. These efforts will advance reliable solutions for smart city surveillance and automated drone inspection.

## Figures and Tables

**Table 1:** Comparison of inference results with other models on the VisDrone2019 test dataset.

Model	mAP@50	mAP@50:95	Parameters	Inference Speed
Yolov8n (baseline)	0.291	0.167	3,007,598	10.8 ms
Yolov10n	0.287	0.162	2,267,118	13.8 ms
Yolov11n	0.292	0.166	2,584,102	14.3 ms
Yolov12n	0.293	0.167	2,558,678	20.8 ms
RT-DETR	0.223	0.117	42,781,282	44.2 ms
RT-DETR-V4-S	0.377	0.216	10,231,995	33.2 ms
Mamba-YOLO	0.314	0.179	5,987,302	47.9 ms
DEIM	0.313	0.173	3,737,611	38.6 ms
ours-x	0.417	0.248	63,072,178	37.7 ms
ours-l	0.409	0.245	40,416,482	25.4 ms
ours-m	0.399	0.238	22,841,218	19.7 ms
ours-s	0.373	0.217	9,758,850	16.4 ms
ours-n	0.326	0.185	2,626,194	15.9 ms

**Table 2:** Inference results for specific categories in the VisDrone2019 test dataset.

Model	Pedestrian	People	Bicycle	Car	Van	Truck	Tricycle	Awning-Tricycle	Bus	Motor	All
Yolov8n	0.238	0.13	0.063	0.681	0.317	0.367	0.165	0.17	0.531	0.243	0.291
Yolov10n	0.237	0.145	0.071	0.682	0.322	0.334	0.145	0.161	0.524	0.25	0.287
Yolov11n	0.246	0.132	0.068	0.684	0.328	0.345	0.165	0.171	0.529	0.253	0.292
Yolov12n	0.243	0.124	0.077	0.692	0.324	0.347	0.161	0.175	0.523	0.261	0.293
RT-DETR	0.179	0.086	0.006	0.659	0.27	0.245	0.088	0.077	0.42	0.19	0.223
RT-DETR-V4	0.316	0.251	0.179	0.767	0.401	0.430	0.241	0.199	0.618	0.366	0.377
Mamba-YOLO	0.258	0.138	0.090	0.714	0.368	0.377	0.167	0.185	0.561	0.288	0.314
DEIM	0.213	0.173	0.108	0.709	0.366	0.381	0.179	0.168	0.559	0.282	0.313
ours-s	0.345	0.223	0.125	0.768	0.406	0.437	0.23	0.223	0.608	0.353	0.373
ours-n	0.307	0.192	0.095	0.734	0.357	0.362	0.175	0.187	0.551	0.3	0.326

**Table 3:** Ablation experiment results for the detection head.

Model	mAP@50	mAP@50:95
baseline	0.291	0.167
+P2	0.297	0.168
+P2-P5	0.308	0.176
+P2-P5-P4	0.305	0.172

**Table 4:** Neck Ablation Experimental Results.

Model	mAP@50	mAP@50:95
baseline	0.308	0.176
BiFPN	0.3	0.17
FPN	0.314	0.174
SFPN	0.322	0.183

**Table 5:** Ablation experiment results for the attention module.

Model	mAP@50	mAP@50:95
baseline	0.322	0.183
C2fA (all layer)	0.324	0.183
C2fA (Shallow layer)	0.321	0.183
C2fA (Deep layer)	0.326	0.185

**Table 6:** YOLO-UTD Model Ablation Experimental Results.

Baseline	Head	Neck	Backbone	mAP@50	mAP@50:95	Parameters	Inference Speed
√				0.291	0.167	3,007,598	10.8 ms
√	√			0.308	0.176	2,010,542	9.5 ms
√	√	√		0.322	0.183	2,594,302	13.2 ms
√	√	√	√	0.326	0.185	2,626,194	15.9 ms

**Table 7:** Experimental Results of Performance Comparison and Improvement Rates Across Models of Different Scales.

Model	Improvement	mAP@50	Performance	mAP@50:95	Performance
Yolov8n		0.29	+12.41%	0.166	+11.45%
Yolov8n	√	0.326		0.185	
Yolov8s		0.329	+13.37%	0.194	+11.86%
Yolov8s	√	0.373		0.217	
Yolov8m		0.363	+9.92%	0.217	+9.68%
Yolov8m	√	0.399		0.238	
Yolov8l		0.382	+7.07%	0.23	+6.52%
Yolov8l	√	0.409		0.245	
Yolov8x		0.385	+8.31%	0.233	+6.44%
Yolov8x	√	0.417		0.248	

**Table 8:** Comparison on UAVDT test set.

Model	mAP@50	mAP@50:95	Parameters
Yolov8n	0.306	0.183	3,006,233
YOLO-UTD	0.382	0.224	2,591,593

## Data Availability

The raw and processed data presented in this study are available on reasonable request from the corresponding author, Meng Li, or the first author, Hailang Huang.
